# Can Italian Healthcare Administrative Databases Be Used to Compare Regions with Respect to Compliance with Standards of Care for Chronic Diseases?

**DOI:** 10.1371/journal.pone.0095419

**Published:** 2014-05-09

**Authors:** Rosa Gini, Martijn J. Schuemie, Paolo Francesconi, Francesco Lapi, Iacopo Cricelli, Alessandro Pasqua, Pietro Gallina, Daniele Donato, Salvatore Brugaletta, Andrea Donatini, Alessandro Marini, Claudio Cricelli, Gianfranco Damiani, Mariadonata Bellentani, Johan van der Lei, Miriam C. J. M. Sturkenboom, Niek S. Klazinga

**Affiliations:** 1 Agenzia regionale di sanità della Toscana, Florence, Italy; 2 Department of Medical Informatics, Erasmus Medical Center, Rotterdam, The Netherlands; 3 Società italiana di medicina generale, Florence, Italy; 4 Genomedics, Florence, Italy; 5 Unità Locale Socio Sanitaria Padova, Padua, Italy; 6 Azienda Sanitaria Provinciale Ragusa, Ragusa, Italy; 7 Assessorato Politiche per la Salute, Bologna, Italy; 8 Zona Territoriale Senigallia, Senigallia, Italy; 9 Università Cattolica del Sacro Cuore, Rome, Italy; 10 Agenzia Nazionale per il Servizi Sanitari Regionali, Rome, Italy; 11 Academic Medical Center, University of Amsterdam, Amsterdam, The Netherlands; S.G. Battista Hospital, Italy

## Abstract

**Background:**

Italy has a population of 60 million and a universal coverage single-payer healthcare system, which mandates collection of healthcare administrative data in a uniform fashion throughout the country. On the other hand, organization of the health system takes place at the regional level, and local initiatives generate natural experiments. This is happening in particular in primary care, due to the need to face the growing burden of chronic diseases. Health services research can compare and evaluate local initiatives on the basis of the common healthcare administrative data.However reliability of such data in this context needs to be assessed, especially when comparing different regions of the country. In this paper we investigated the validity of healthcare administrative databases to compute indicators of compliance with standards of care for diabetes, ischaemic heart disease (IHD) and heart failure (HF).

**Methods:**

We compared indicators estimated from healthcare administrative data collected by Local Health Authorities in five Italian regions with corresponding estimates from clinical data collected by General Practitioners (GPs). Four indicators of diagnostic follow-up (two for diabetes, one for IHD and one for HF) and four indicators of appropriate therapy (two each for IHD and HF) were considered.

**Results:**

Agreement between the two data sources was very good, except for indicators of laboratory diagnostic follow-up in one region and for the indicator of bioimaging diagnostic follow-up in all regions, where measurement with administrative data underestimated quality.

**Conclusion:**

According to evidence presented in this study, estimating compliance with standards of care for diabetes, ischaemic heart disease and heart failure from healthcare databases is likely to produce reliable results, even though completeness of data on diagnostic procedures should be assessed first. Performing studies comparing regions using such indicators as outcomes is a promising development with potential to improve quality governance in the Italian healthcare system.

## Introduction

Primary care is specifically suitable to face the growing chronic disease epidemic in a sustainable way [1], [2]. Therefore it is the object of novel attention and of innovative policies [3] which specifically need health services research for timely effectiveness evaluation [4]–[7].

Many observational studies have been performed to evaluate the impact of innovative policies in primary care, for instance alternative rewarding policies for General Practitioners (GPs) in Ontario [8] or incentives for the introduction of Electronic Health Records in the United States [9], [10]. Such studies use administrative data to obtain evidence on the impact of policies in a inexpensive, timely and reproducible fashion [11]. Indicators measuring compliance with standards for management of chronic diseases were used as outcomes in those studies, similar to the clinical indicators of the Quality and Outcome Framework of the UK National Health System [12], such as regular prescription of recommended therapies and regular diagnostic follow-up. However, concerns have been raised that such indicators estimated on the basis of administrative databases might not reflect the actual compliance of standards in the population bearing the disease, as methods to identify patients from administrative data, rather than clinical information, might lead to biased samples. Studies addressing this issue have obtained contradictory findings [13], [14].

As a result of those concerns, comparison of quality of primary care between regions or countries is generally performed by means of hospitalization rates for the so-called ambulatory care sensitive conditions [15], which are readily obtained from administrative databases but do not require identification of cohorts of patients with a specific condition. However the relationships between quality of primary care and avoidable hospitalization is complex and population-based trends can be confounded by socioeconomic factors [16], by prevalence of morbidity or general hospitalization habits [17].

In Italy, the VALORE Project was the first national-level study which evaluated a national policy in primary care by using administrative healthcare data for calculation of indicators of compliance [18]. This paper presents the validation study on the reliability of administrative databases in estimating such indicators.

## Materials and Methods

### Ethics

No identifiable human data were used for this study. The dataset used in the study is not openly available. According to the Italian law on data confidentiality (decree 196/2003), permission to use individual-level data, albeit non-identifiable, must be granted by the institutions which bear the responsibility of the custody of the data. Permission to use data extracted from administrative databases for the VALORE project was granted to Agenzia regionale di Sanità della Toscana by ULSS 16 Padova (Veneto region), ASP 7 Ragusa (Sicily region), Assessorato Politiche per la Salute Emilia Romagna (Emilia Romagna region), Zona Territoriale Senigallia (Marche region), which are responsible for the custody of the data of the corresponding populations. Agenzia regionale di sanità della Toscana (Tuscany region) is enabled by a regional law (40/2005) to use Tuscan data for research purposes. Approval for use of encrypted and aggregated data from the HSD was also obtained from the Italian College of General Practitioners.

### Setting

Italy has a tax-based, universal coverage national health system organised in three levels: national; regional (21 regions); and local (on average 10 Local Health Authorities per region). Healthcare is managed for every inhabitant by the Local Health Authority where she has her regular address [19]. Coordination of primary care within a Local Health Authority is performed at a smaller geographical level called Health District [18]. Every Italian inhabitant is entitled to choose a GP, although parents might opt for a specialist paediatrician instead for their children, up to the age of 15. Therefore, each inhabitant from the age of 16 onward is specifically associated with a GP. GPs are the “gatekeepers” of the system, meaning that only upon GP prescription can specialist encounters be obtained free of charge. Dispensing of drugs or administration of diagnostic procedures can be obtained free of charge upon prescription of either a GP or a specialist physician employed by the healthcare system [19].

The five regions which contributed data to the VALORE validation study were: Veneto (A, Northern Italy), Emilia Romagna (B, Northern Italy), Tuscany (C, Central Italy), Marche (D, Central Italy) and Sicily (E, Southern Italy).

### Study design

The VALORE project had selected several indicators to measure compliance with standards of care for diabetes, IHD and HF. In each region from the pool of regional GPs two convenience samples of groups of GPs were extracted and included in the validation study. In each regional pair, GPs of one sample had indicators computed from administrative databases, GPs of the other from their own clinical databases. Measurements of indicators were compared within and between regions.

The true values of an indicator across all the GPs in a region are an unobservable distribution. The rationale of this study design is based on the assumption that if measurements performed with two different methods in two different samples of GPs provide similar results, the likelihood that they both measure the true distribution is higher than the likelihood that they systematically make the same mistakes across different regions.

### Data collection: sample of GPs with administrative-based measures

The national Italian government has mandated since the early Nineties collection of healthcare administrative data across the whole country. The healthcare activities which are mandated to be reported to the government have progressively expanded, from inpatient care [20] to drug dispensings and diagnostic tests [21]. Moreover an inhabitant registry is maintained by each Local Health Authority, where the GP chosen by the inhabitant is recorded, as well as other information, such as gender, birth date, date of entry in the territory of the Local Health Authority, date of exit from the territory [21]. However, outpatient diagnoses are not recorded in health administrative databases yet. Therefore cohorts of patients with chronic diseases must be selected by means of disease-specific longitudinal algorithms involving hospital discharges diagnoses, drug and/or other healthcare services utilization.

In each region, a convenience sample of Health Districts was chosen. All the GPs serving in those Health Districts were identified from the inhabitant registries of the corresponding Local Health Authorities and included in the sample. The healthcare administrative data of the whole population who chose a GP in this sample was loaded in the VALORE database. Patients aged 16–95 with diabetes, IHD and/or HF at the index date 1/1/2009 were detected by means of ad hoc algorithms based on past healthcare received. More details on this process are described elsewhere [22]. Indicators were computed during a one-year follow-up by linking the cohorts to the administrative databases of drug dispensings and diagnostic tests.

GPs were excluded from the samples if they had less than 300 persons registered or less than 4 patients with the disease, as indicators computed on small numbers were considered to be non robust.

### Data collection: sample of GPs with clinical-based measures

The samples of GPs with clinical-based measures were drawn from the Health Search CSD Longitudinal Patient Database (HSD), a longitudinal observational database that is representative of the general Italian population. HSD was established in 1998 by the Italian College of General Practitioners and, at the time when the study was conducted, it contained data from from more than 800 GPs throughout Italy, covering a total population of around 1.2 million patients [23]. The GPs participating in HSD all use the same information software, in which they record demographic information, visits and referrals, diagnoses, drug and diagnostic tests prescriptions and clinical information of their patients. They are accepted as participants in HSD if their records are arguably complete, i.e. the prevalence of the principal diseases measured from their records is comparable with the expected prevalence of the general population. For this study, data from the 190 GPs practicing in the five regions of the VALORE project were used. The study population comprised patients aged 16-95 who had been registered with the GP for at least two years and were alive on 1st January 2009. Patients with diabetes, IHD and/or HF at the index date 1/1/2009 were detected by means of algorithms based on recorded diagnosis, which is described in detail elsewhere [22]. Indicators were computed from the prescribed drugs or diagnostic tests.

### Indicators

The indicators that were included in the study are shown in Table 1, and are classified as therapy indicators (for IHD and HF only), laboratory diagnostic tests, and bio-imaging diagnostic tests (HF only). All the indicators were based on clinical guidelines for the management of the disease that recommended regular therapy and yearly testing, respectively. The standard for a therapy recommendation was considered to be compliant with when at least two dispensings (in VALORE) or prescriptions (in HSD) were recorded in 2009, at least 180 days the one from the other. The standard for a diagnostic recommendation (laboratory or bioimaging) was considered to be achieved when at least one test was performed (in VALORE) or prescribed (in HSD) during 2009.

**Table 1:** List of indicators.

	Therapy (≥2 dispensations per year, distance >180 days)	Laboratory diagnostic test (≥1 per year)	Imaging diagnostic test (≥1 per year)
Diabetes		Creatinine, Glycated emoglobin	
IHD	ACE inhibitors, Antithrombotics	Total colesterol	
HF	ACE inhibitors, Beta-blockers		Ecocardiogram

Indicators for the care of chronic diseases selected by the VALORE project and included in the validation study.

### Statistical analysis

In each sample the number of GPs, the number, age and gender distribution of patients aged 16+, and the average number of patients per GP were computed, both for the general population and for the population with each of the diseases. Differences in the variables within each regional pair of samples were tested either by a two-tail difference in means or a Chi-square test.

For each GP indicators were computed as percentage of patients who were compliant with the recommended standards of care. The distribution of the indicators of each regional pair were represented in a box-plot. To test whether each pair of measurements was drawn from the same distribution, the non-parametric Wilcoxon-Mann-Whitney two-sample statistic (also known as Wilcoxon rank-sum statistic) was performed in each region and for each indicator. In a sensitivity analysis, the test was repeated for achievements of standards in patients aged 45–74 years.

Data management and data analysis were performed with Stata 10.1.

## Results

Of the 1671 GPs serving in the Health Districts participating to the VALORE study, 1501 (89.8%) had enough registered patients and entered the study. Few GPs were discarded from the disease-specific studies because they had less than four patients, the maximum was the 7% of GPs in region A in the HF study. All the 190 GPs in the HSD sample entered the study.

The description of the study population is shown in Table 2. Every HSD sample contained less GPs than the VALORE sample. The GPs in the HSD sample had a bigger registered population on average in all the five samples (range in HSD: 1238–1431, range in VALORE: 925–1223). The average number of patients per GP was higher in HSD GPs as well for diabetes (range in HSD 92.0–107.5, range in VALORE: 55.9–81.6) and IHD (range in HSD: 50.8–78.6, range in VALORE: 40.0–61.9), but for HF the numbers were similar in the two groups (range in HSD: 13.7–22.2, range in VALORE: 12.8–20.0). Age distribution was different within all pairs in all the populations, and the VALORE samples were older except in region B. Women were slightly more represented in the VALORE populations, except again in region B and in region E. This difference in gender did not show up in diabetic patients and was not consistent across regions in IHD and HF patients.

**Table 2:** Study population.

			A			B			C			D			E		
			HSD	VALORE	p	HSD	VALORE	p	HSD	VALORE	p	HSD	VALORE	p	HSD	VALORE	p
General population	N GP		50	140		38	619		28	484		17	56		57	202	
	N population		67314	156516		54384	757163		34666	447577		23439	55992		75299	220843	
	N registered per GP		1346.3	1118.0	<0.001	1431.2	1223.2	<0.001	1238.1	924.7	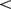 0.001	1378.8	999.9	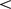 0.001	1321.0	1093.3	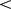 0.001
	Female		52.7	54.5	<0.001	53.6	51.4	<0.001	52.5	54.2	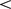 0.001	51.1	52.0	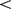 0.05	52.8	50.9	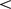 0.001
	Ageband	16–44	42.7	37.9	<0.001	37.4	42.3	<0.001	38.8	33.4	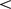 0.001	43.5	35.6	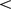 0.001	47.7	43.7	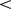 0.001
		45–64	32.4	34.5		32.4	31.5		32.4	35.6		31.7	33.9		30.9	32.2	
		65–74	13.0	14.2		14.7	12.9		14.2	15.9		11.8	15.0		10.9	12.4	
		75–84	8.8	9.8		11.1	9.6		10.7	11.4		9.4	11.4		8.0	8.9	
		85–95	3.0	3.6		4.3	3.7		4.0	3.8		3.7	4.1		2.4	2.8	
Diabetes	N GP		50	137		38	619		28	484		17	56		57	202	
	N patients		4986	9122		4085	39138		2577	28872		1793	3132		7095	16490	
	N registered per GP		99.7	66.6	<0.001	107.5	63.2	<0.001	92.0	59.7	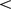 0.001	105.5	55.9	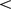 0.001	124.5	81.6	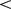 0.001
	Female		45.2	46.7	0.089	46.0	47.1	0.193	48.3	49.3	0.313	46.4	47.5	0.454	51.4	50.8	0.388
	Ageband	16–44	5.2	6.4	<0.001	3.6	5.7	<0.001	4.0	5.2	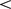 0.001	5.6	5.2	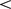 0.05	7.0	4.3	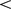 0.001
		45–64	32.0	25.5		29.2	27.3		31.8	26.6		31.7	26.8		37.0	30.5	
		65–74	32.3	30.4		31.4	28.7		30.4	30.1		28.7	29.7		27.9	29.9	
		75–84	22.8	27.2		26.9	27.1		25.9	28.5		24.7	27.6		22.7	26.9	
		85–95	7.7	10.6		8.9	11.2		7.9	9.6		9.4	10.8		5.5	8.4	
IHD	N GP		50	137		38	619		28	484		17	56		57	201	
	N patients		2538	6041		2985	38324		1584	19366		1070	2622		3581	9782	
	N registered per GP		50.8	44.1	<0.05	78.6	61.9	<0.001	56.6	40.0	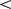 0.001	62.9	46.8	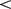 0.001	62.8	48.7	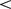 0.001
	Female		38.1	41.9	<0.05	40.4	45.2	<0.001	39.4	42.0	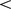 0.05	40.7	39.9	0.669	39.6	40.0	0.657
	Ageband	16–44	2.2	1.1	<0.001	1.1	1.1	<0.001	1.1	0.9	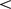 0.001	1.3	1.1	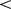 0.001	3.2	1.8	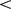 0.001
		45–64	22.5	15.4		20.3	15.3		18.9	16.0		23.5	15.5		28.3	19.9	
		65–74	31.0	24.1		29.7	23.0		28.6	25.6		28.8	23.8		30.0	26.8	
		75–84	31.8	35.9		34.1	36.3		35.3	36.9		32.2	36.2		29.0	34.9	
		85–95	12.5	23.5		14.8	24.4		16.1	20.7		14.2	23.4		9.5	16.5	
HF	N GP		47	130		38	605		26	479		17	54		53	197	
	N patients		664	2110		842	12107		395	6577		296	692		726	2794	
	N registered per GP		14.1	16.2	0.080	22.2	20.0	0.150	15.2	13.7	0.229	17.4	12.8	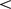 0.05	13.7	14.2	0.642
	Female		45.8	55.4	<0.001	56.3	51.8	<0.05	50.6	45.7	0.058	50.7	50.7	0.989	53.7	48.0	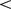 0.05
	Ageband	16–44	0.5	0.7	<0.001	0.8	1.1	0.504	1.3	1.1	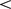 0.001	1.4	0.3	0.253	1.7	2.0	0.671
		45–64	13.0	7.4		10.2	9.0		8.4	13.5		10.1	8.8		13.1	12.6	
		65–74	21.4	15.5		18.5	17.4		17.7	22.2		18.2	16.3		20.5	22.3	
		75–84	37.5	37.8		38.5	38.5		40.0	37.7		39.9	41.2		42.1	39.7	
		85–95	27.7	38.6		31.9	34.0		32.7	25.4		30.4	33.4		22.6	23.3	

Description of the GPs in the five (A, B, C, D, E) pairs of samples (HS, measured by means of clinical data, and VALORE, measured by means of healthcare administrative data). Description of the general population they have in charge (general population), of diabetic patients (Diabetes), of patients with ischaemic heart disease (IHD) and of patients with heart failure (HF). N GP: number of GPs in the study. N populations: number of inhabitants registered with the GPs in the study. N patients: total number of patients with the chronic disease registered with GPs in the study. N registered per GP: average number of persons in charge to each GP, with test for difference in means within each pair. Female: percentage of women in the population, with test for difference in means within each pair. Age band: age distribution of the population, with chi square test within each pair.

**Figure 1:**
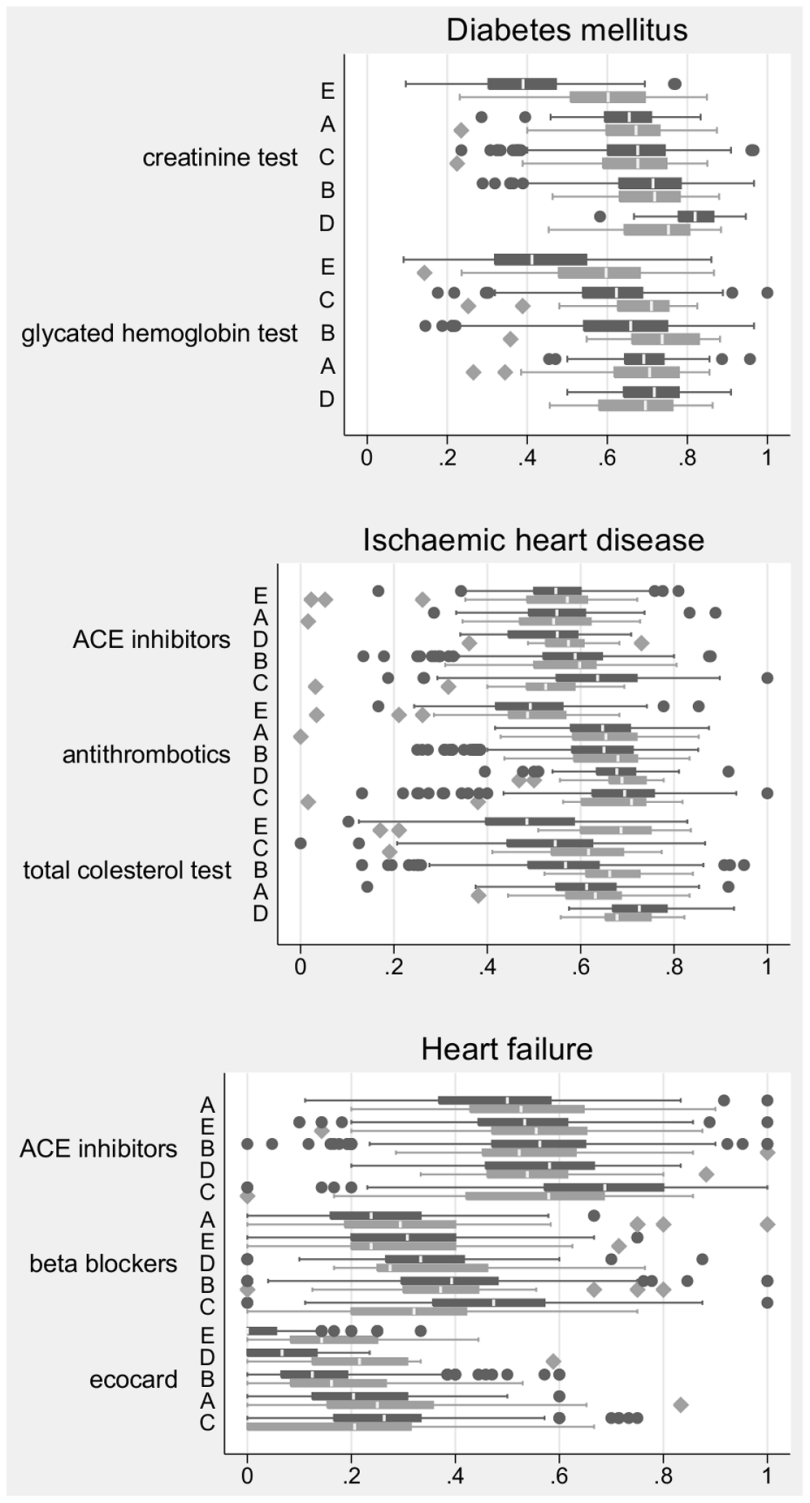
Box-plots of the distribution of indicators of quality of care for diabetes (2 indicators), IHD (3 indicators) and HF (3 indicators) in 5 pairs of samples of GPs. Each pair contains the distribution obtained from the VALORE data (dark gray) and the distribution obtained from HSD data (light gray). For each indicator the pair of samples are ordered according to the median value in the VALORE sample.

Figure 1 shows the box-plots of the pairs of distributions of the crude values of each indicator. A qualitative examination of the box-plots detected that distributions are very similar within the pairs. A notable exception are laboratory measurements in region E and bio-imaging test in all the regions, and VALORE showed lower values in all cases. The geographical trends, represented by orderings of the median values of the distributions, were similar between regions when measured in either data source, but less so in the case of the bio-imaging test.

Table 3 shows the results of the Wilcoxon-Mann-Whitney tests. Among therapy indicators the test found no differences in the distributions, with the exception of the samples in region C and, for HF only, region A, and the VALORE samples had higher values. The test confirmed that the distributions for all the laboratory diagnostic indicators of region E were different. Among diabetes the test detected slightly different distributions in three regions in either of the indicators, and in the IHD indicator region C and B had different distributions. The test confirmed that the only indicator of bio-imaging testing resulted in incoherent measurements in all but one region. Restricting the distributions to age-specific indicators (45–74) improved the comparability of the distributions of the therapy indicators of HF, and left unchanged the comparability of the other indicators.

**Table 3:** P-values of the Wilcoxon-Mann-Whitney tests.

Disease	Indicators	Region	Pooled	45–74
Diabetes	Creatinine test	A	0.357	*
		B	0.587	0.701
		C	0.840	0.957
		D	*	*
		E	**	**
	Glycated emoglobin test	A	0.628	0.653
		B	**	*
		C	*	*
		D	0.441	0.441
		E	**	**
Ischaemic heart disease	ACE inhibitors	A	0.728	0.067
		B	0.695	0.671
		C	**	**
		D	0.116	0.065
		E	0.504	0.877
	Antithrombotics	A	0.508	0.174
		B	0.328	0.084
		C	0.651	0.440
		D	0.497	0.588
		E	0.754	0.095
	Total cholesterol test	A	0.225	0.962
		B	**	**
		C	*	**
		D	0.279	0.720
		E	**	**
Heart failure	ACE inhibitors	A	*	0.960
		B	0.454	0.107
		C	*	*
		D	0.701	0.961
		E	0.267	0.052
	Beta-blockers	A	*	0.670
		B	0.389	0.490
		C	**	0.091
		D	0.914	0.523
		E	0.293	0.614
	Ecocardiogram	A	0.134	0.245
		B	*	*
		C	0.059	0.944
		D	**	**
		E	**	**

P-values of the Wilcoxon-Mann-Whitney tests. P-values smaller than 0.05 are represented by a single star, P-values smaller than 0.001 are represented by a double star.

## Discussion

Even though in Italy the data items to be collected in health administrative databases are mandated by the central government, and the resulting central databases are therefore formally homogeneous, data collection takes place locally. Italy is characterized by long-standing regional differences in general and in healthcare in particular [24]. Therefore it is possible that inaccurate local data collection processes hamper data quality and completeness, and in particular quality of personal identifiers that allow for record-linkage. Moreover, outpatient diagnosis are not among the data items collected, therefore identification of cohorts of patients with chronic diseases must rely on algorithms linking inpatient diagnosis with drug and other healthcare services utilization. Inhomogeneous quality of personal identifiers and completeness of recordings might lead to inhomogeneous accuracy in defining cohorts of patients and in identifying healthcare services that they access. This in turn might result in non-comparable measures of compliance with standards of care for chronic diseases.

This study addressed this concern by comparing such measures with measures obtained from a different data source, in five Italian regions. The database which was chosen as a comparator collects clinical data from GPs, and is therefore complementary to the healthcare administrative data.

The results show that administrative databases provide reliable estimates on regional level. Indeed, the four therapy indicators had the same distribution within the pairs of regional samples in the large majority of cases. The same was observed for the three diagnostic indicators except in one region, where the distributions were systematically different. The only bio-imaging indicator had different distributions within pairs. Geographical trends between regions were consistent across the two data sources. This provides evidence that the two data sources both estimate the same population distribution, thus supporting the use of indicators computed on health administrative databases for comparisons between regions.

It was not possible to obtain measurements from the two data sources on the same samples of GPs. This was partly due to the fact that the identity of the GPs belonging to the database HSD is confidential. Moreover, data linkage at individual or even GP level between different data sources had legal implications in terms of privacy regulations and the procedures needed to obtain permissions for such data collection [25] could not be managed in the context of the VALORE Project.

Therefore, observed differences in the distributions might be attributable to the composition of the following main effects: (a) due to non-random selection of the two samples, the GPs in the two samples were qualitatively different with actually different performance; (b) due to the different selection process that was conducted in the two type of data sources, the cohorts of patients of the two samples were qualitatively different subpopulations of the actual patients, which actually received different care; (c) difference in measurement, and HSD was likely biased (d) difference in measurement, and VALORE was likely biased. In the following paragraphs we provide plausible explanations to disentangle the effect (d), which is the object of this study, from (a), (b) and (c). It is a limitation of this study (see Limitations subsection) that some of the hypotheses we generated could not be tested. For cause (b), the main reference is the study by Gini et al, which found evidence that diabetic patients without therapy are less prevalent in the VALORE sample, and patients with heart failure are younger in the GP sample [22].

For therapy indicators some differences are observed for HF in regions A and C. This is most likely due to reason (c), that is, patients included in the cohorts of HSD samples are different than patients included in the cohorts of the VALORE samples: indeed, age distribution of patients is different within the pairs, with the older cohort in VALORE being more likely to be assisted at home or in residential facilities, where GPs are likely to not record their activity completely [22], [23]. To test this assumption, analysis was restricted to patients aged 45–74, and indeed differences disappeared in region C in one indicator and in region A in both.

For laboratory testing indicators, region E seem to underestimate consistently the actual values of the indicators, across the three diseases. This could amount to incomplete collection of administrative data from laboratories, or to higher use of out-of pocket services: indeed, the most recent National Health Survey found that in region E (Sicily) attitude to use diagnostic services that are non reimbursed by the Health System is higher than in the other regions participating to our study [26]. In the other regions differences do not show a consistent pattern, except perhaps in region C, where however (a) rather than (d) could be the cause, that is, GPs in the HSD sample and GPs in the VALORE sample in region C actually have different quality of care. Indeed, in region C therapy indicators differ slightly between samples as well.

The bio-imaging indicator is probably underestimated by healthcare administrative databases: this might be due to out-of-pocket payment of this analysis, or to the fact that bio-imaging occurring during hospital admissions was not recorded by VALORE.

The overall similarity in measurements that was observed in this study generates in turn two observations. First, the standards of care in the sample of GPs participating to the HSD database seem to be representative of the distribution of the whole population of GPs. This was surprising, as GPs in HSD are selected because of completeness in their recordings, and good recording habits were expected to be associated with better standards of care. The second observation is that specialist physicians who assist chronic patients are likely to involve GPs in regular prescription of therapies and diagnostic tests: indeed, if GPs were unaware of such prescriptions in the share of patients who are visited by a specialist, their clinical data would detect lower standards.

Our study was performed in samples drawn from regions belonging to three macro-areas of the country. Only a study performed in all regions could rule out the possibility that major issues show up in other areas, however the evidence we observed points to the direction of greater confidence. On the other hand, we do not claim that our results support reliability of similar measurements for any chronic disease. Indeed, this is determined by how reliable the algorithm for identifying the case is, and it was shown that this depends specifically on the disease, as frequency of hospital use, specificity of drug indication and pattern of healthcare may vary [22].

In summary, the evidence we provide is promising enough to support comparison of regions with respect to indicators of compliance with standards of care for diabetes, IHD, and HF. Moreover, it supports the reliability of empirical studies, as the VALORE study [18], using such indicators to evaluate the impact of organizational innovation in primary care.

### Limitations

In this study indicators were computed on a population level for a convenience samples of GPs instead of directly being compared on a patient level for the same GPs. Similarity between samples could be due to random combination of contrasting effects rather than being attributable to the factors that we discussed. Although this is unlikely to have happened consistently in five regions, an individual-level validation study only could address this concern. Italy, like several other countries, has a national legislation that permits exemption to the requirement for patient consent for projects in the public's interest [25], but this pathway was too complex to be faced in the context of the VALORE project.

It was not possible to test some of the hypotheses we generated to explain observed differences. A study involving more regions and different points in time could provide counterfactuals to test our hypotheses.

## Conclusion

According to the evidence presented in this study, estimating compliance with standards of care for diabetes, ischaemic heart disease and heart failure from healthcare databases is likely to produce reliable results, even though completeness of data on diagnostic procedures should be assessed first. Performing studies comparing regions using such indicators as outcomes is a promising development with the potential to improve quality governance in the Italian healthcare system.
